# Consequences and management of neck pain by female office workers: results of a survey and clinical assessment

**DOI:** 10.1186/s40945-016-0023-3

**Published:** 2016-07-18

**Authors:** Venerina Johnston

**Affiliations:** School of Health and Rehabilitation Sciences, The University of Queensland, Brisbane, Australia

**Keywords:** Neck problem, Pain management, Public health

## Abstract

**Background:**

Neck pain is common in office workers. However, the functional consequences of this pain to the individual and how they are managed are not well known. The objective of this study is understand the impact of neck pain and the strategies female office workers use to manage their pain while remaining at work.

**Methods:**

Female office workers with neck pain (*n* = 174) completed a survey about the impact of their neck pain, with 51 attending a university clinic for further assessment. Consequences of neck pain were evaluated with questions on self-reported work absence, workers’ compensation claims, health care use, impact on work and leisure activity, and management strategies. Responses to survey questions were analysed using descriptive analyses.

**Results:**

The results showed that during the preceding 12 months, 57.5 % of participants had consulted a health professional due to neck pain; 42 % had reduced their leisure activities; 22.4 % had reduced their work activity and 20.7 % had been absent from work. Only 5.2 % had ever submitted a workers’ compensation claim and 9 % indicated changing jobs due to neck pain. Of the 51 participants who attended for further assessment, 35.3 % indicated they ‘self-managed’ their neck pain with conventional medical strategies. Common strategies utilized were: prescription or over-the-counter medications (82.5 %), physiotherapy (64.7 %) and visiting their general medical practitioner (54.9 %).

**Conclusions:**

Although the severity of neck pain experienced by female office workers in this study was low, the impact on work and leisure was substantial. These workers tended to self-manage their pain by reducing work and/or leisure activity and utilizing passive coping strategies to remain at work. Physiotherapists are ideally suited to provide self-management strategies to ensure workers remain healthy while working.

## Background

Neck pain is a common problem in the working population [1, 2]. In particular, the prevalence of neck pain in office workers has been reported to be between 50 and 76 % in Australia and 45–63 % internationally [3–8]. Despite this, little is known about the consequences of this problem to the individual office worker and which strategies, if any, are utilized to ensure they remain at work [4]. Evidence suggests that neck pain may lead to care seeking behaviour, sickness absences and workers’ compensation claims [4, 9, 10]. In Australia, neck pain account for a small proportion of all serious workers’ compensation claims (2.2 %) [11], yet the contribution of persistent neck pain to the total burden of chronic pain in Australian society is 20 % [12]. Neck pain accounts for 20 % of the $34 billion each year spent on chronic pain in the Australian community [13]. Thus, gaining a greater understanding of how office workers manage their pain can enhance the development of validated and cost effective interventions and reduce the burden on the individual and the employer.

Current strategies utilized by office workers to remain at work with neck pain are unknown. Exploring the coping strategies office workers employ to manage their neck pain may provide some insight into the importance of this problem and help direct interventions in the workplace. A recent study of patients’ experience and management of neck pain in general practice found that many self-managed their pain with techniques like massage and “over-the-counter medication” [14]. However, this study was conducted in Germany, which has a different health care system than Australia, where conservative interventions such as physiotherapy must be prescribed by a medical practitioner. Furthermore, it was not specifically conducted in a working population. The aims of this study were to 1) describe the consequences of neck pain for female office workers and 2) explore their self-reported management and coping strategies. It was hypothesised that female office workers with neck pain generally, do not submit worker’s compensation claims but remain in the workforce by managing symptoms at the individual level by taking sick leave, visiting a health care professional and self-medication.

## Methods

### Study design

Data from eligible female office workers was obtained through a cross-sectional survey and clinical assessment. This research focused on neck pain in female office workers as females consistently demonstrate an increased prevalence of neck disorders and are usually over-represented in the office worker population [5, 15].

### Participants

Office workers with neck pain over the age of 18 years and working at a computer more than 20 h per week were invited from 12 public and private institutions in the banking, local government and health industry sectors. A total of 333 office workers volunteered (overall response rate of 30 %) and were screened for eligibility based on the severity of neck complaints and history of neck trauma [3]. Those scoring greater than 8 % on the Neck Disability Index (NDI) and free of neck trauma were deemed as having neck complaints (*N* = 174, response rate 52.3 %). The score on the NDI was selected as the cut-off as this severity is indicative of mild to severe neck problems [16]. History of neck trauma was established by one question as previous musculoskeletal trauma of the neck, shoulder or arm has been shown to be a significant predictor of work absence [17]. The characteristics of all employees were established through an employee profile provided by each organization including age, gender, job titles, employment status, hours worked per week, and type of work performed. This profile did not differ between participants and non-participants.

### Procedure

**Fig. 1:**
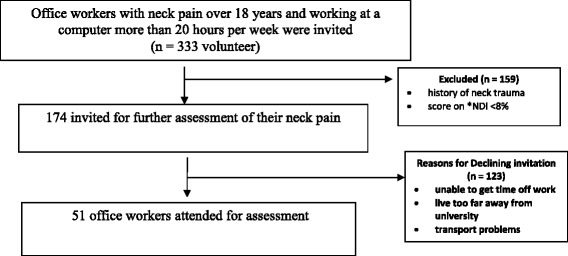
Flow chart of participants through the study. *NDI Neck Disability Index

The comprehensive online survey collected demographic data and information about the consequences of their neck problem on their work and home activities, workplace psychosocial demands and physical ergonomic demands of their work [3]. The 174 eligible office workers were invited to attend a university clinic for further assessment of their neck pain of which 51 participants (29 %) agreed. To limit selection bias, no financial incentives or offers of treatment were provided. Figure 1 demonstrates the flow of participants in the study. The time lapse between survey and assessment was on average, 1 month. The assessment was undertaken during the worker’s lunch time or after hours and consisted of an interview and physical examination performed by a qualified physiotherapist. The purpose of the interview was to further understand and describe the strategies used to manage their pain and consisted of questions about the severity and history of pain and the type of management sought. The physical examination consisted of manual palpation of the neck, assessment of active physiological movements of the neck and shoulders.

### Ethical considerations

All participants were informed that participation was voluntary and that no remuneration or incentives would be provided by the investigators or their employer. Information on individual results was not released to the employer. Ethics for this study was granted by The University of Queensland Medical Ethics Committee (#2004000225).

### Measures

#### Severity of neck pain

The NDI assessed the severity of disability due to neck pain [16] as this tool has been shown to have good test-retest reliability and internal consistency [18]. This index includes ten items that address functional activities including sleeping, reading, lifting, personal care, recreation, driving and work. There are six possible responses for each item which are scored from 0 (no disability) to 5 (complete disability). The final score is obtained as a percentage after adding the scores for each of the 10 items. A higher score indicates greater pain and disability.

#### Consequences of neck complaints

The Nordic Musculoskeletal Questionnaire (NMQ) was used to determine the duration of neck pain with the question “*What is the total length of time that you have had neck trouble (ache, pain or discomfort) during the last 12 months?”* [19] Five response options were possible from No days, 1–7days, 8–30days, >30 days but not every day, and every day. A body map was included to assist participants to understand the area defined as the neck. This tool is widely used in occupational research [20] and is a validated and reliable tool [21].

Absence from work due to neck pain was assessed by one question from the NMQ: “*Have you been absent from work during the last 12 months because of trouble in the neck?”* This question showed high specificity and sensitivity when used to measure the occurrence of sickness absence due to back pain [22]. It has also been used in the assessment of sickness absence due to neck and upper extremity pain [23].

Health care use was determined by one question from the NMQ, *“Have you been seen by a doctor, physiotherapist, chiropractor or other health professional because of trouble in the neck during the last 12 months?”*. In the Australian health care system, workers do not require a medical referral to consult with allied health practitioners and may self-refer without the knowledge of the workplace or the worker’s general practitioner.

Consequences on the workers’ work activity, leisure activities, and submission of workers’ compensation claims were evaluated with one question each with a dichotomous response option of No/Yes. These questions were from the NMQ [19]:

- *“Have you ever had to change jobs or duties because of neck trouble”*
- *“Has neck trouble caused you to reduce your activity at work during the last 12 months”*
- *“Has neck trouble caused you to reduce your leisure activity during the last 12 months?”*
- *“Have you ever been absent from work during the last 12 months because of neck trouble?”*
- *“Have you ever submitted a worker’s compensation claim because of neck trouble?”*

The reliability of the NMQ to collect data on the prevalence and consequences of musculoskeletal pain has been established and shown to range from moderate to almost perfect [24]. The duration of time that neck pain affected work activity was evaluated with one question “*What is the total length of time that neck trouble has prevented you from doing your normal work (at home or away from home) during the last 12 months*?” with four response options: No days, 1–7 days, 8–30 days or greater than 30 days.

In the interview at the university clinic, participants were asked two-open ended questions: *‘What do you think has caused your neck problems?’* and *‘How do you manage your neck pain?’* The open ended questions were to reduce potential bias in responses. Responses to the first question were used to establish the work-relatedness of neck pain and were grouped into similar concepts with any ambiguity checked by another researcher.

The responses to the second question were used to determine the coping strategies adopted. Coping mechanisms refer to the specific thoughts and behaviours people use to manage their pain or their emotional reactions to their pain [25]. These self-management strategies were classified as either active or passive coping strategies based on the categorization tables of Brown and Nicassio [26]. Blyth et al. [27] subsequently used these groupings to examine the relationship between self-management strategies, disability and health care utilization in a population-based study of individuals with chronic pain. These authors identified passive coping strategies as any treatment where something was done to, or given to the patient. This was further divided into the two sub-categories of passive behavioural (e.g. massage, rest, heat) and conventional medical management strategies (e.g. medication, physiotherapy as these are given to or one to the patient). Active coping strategies were described as any instrumental activity initiated by the individual to manage their pain, if not characterised by avoidance or escape. For example, while rest may be initiated by the individual, it is considered a passive strategy as it is intended to escape from pain rather than to function despite the pain. These strategies were further divided into active behavioural with a physical component (e.g. exercise, postural modification) and cognitive (e.g. relaxation, mental distraction).

### Analysis

Responses to the survey questions were analysed using descriptive statistical analyses to determine the percentage of positive and negative responses to each question about the impact of neck problems. SPSS version 22 (IBM Corporation New York, USA) was used to manage the data. Strategies to manage neck problems reported by the 51 participants were categorised as active (behavioural or cognitive) strategies or passive (behavioural or conventional medical) strategies. The percentage of participants nominating any of these four categories was calculated.

## Results

In the last 12 months, 82 % of the office workers who volunteered reported experiencing neck pain. The mean score on the NDI of all 174 participants was 20.2 % (SD = 9.1) indicating mild neck pain and disability. The median age of participants was between 40 and 44 years. The mean age of the subsample of 51 participants was 44.3 years (SD = 9.5) with a mean NDI score of 22 % (SD = 8.9). This NDI score indicates that those who volunteered for further assessment were representative of the total sample population.

### Survey results

Table 1 displays the reported consequences of neck pain in symptomatic female office workers. The results revealed that 20.7 % of participants who reported neck pain in the last 12 months were absent from work due to this pain. In addition, 14.9 % of workers did not participate in activities of daily living or normal work duties for 8 or more days in the last 12 months due to pain. Nearly a quarter of those surveyed indicated reducing their work activity due to neck pain but the impact on leisure activities was greater with 42 % affected. Only a small proportion of subjects reported changing jobs as a consequence of their neck pain. Advice and/or treatment from a health care professional was sought by 57.5 % of participants with only 5.2 % reported making a workers’ compensation claim in relation to their neck pain.

**Table 1:** Responses to survey questions on the consequences of neck pain (*N* = 174)

Survey question		Percent	(n)
Have you ever had to change jobs or duties because of neck trouble?	No	90.8 %	(158)
	Yes	9.2 %	(16)
Has neck trouble caused you to reduce your activity at work during the last 12 months?	No	77.6 %	(135)
	Yes	22.4 %	(39)
Has neck trouble caused you to reduce your leisure activity during the last 12 months?	No	58.0 %	(101)
	Yes	42.0 %	(73)
Have you ever been absent from work during the last 12 months because of neck trouble?	No	42.5 %	(74)
	Yes	57.5 %	(100)
What is the total length of time that neck trouble has prevented you from doing your normal work (at home or away from home) during the last 12 months?	0 days	54.6 %	(95)
	1-7days	30.5 %	(53)
	8-30days	8.6 %	(15)
	>30 days	6.3 %	(11)
Have you ever been absent from work during the last 12 months because of neck trouble?	No	79.3 %	(138)
	Yes	20.7 %	(36)
Have you ever submitted a worker’s compensation claim because of neck trouble?	No	94.8 %	(165)
	Yes	5.2 %	(9)

### Subsample results (*N* = 51)

**Fig. 2:**
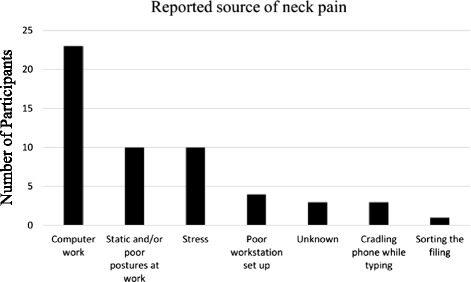
Reported source of neck pain in 51 female office workers

Figure 2 displays the self-reported source of neck symptoms. The majority of participants believed performing computer work including keyboard work, mouse work and reading at computer was the source of their pain. Many participants indicated more than one source of pain.

Table 2 displays the active and passive self-management strategies utilised by the subgroup of female office workers to manage their neck pain. It can be seen that passive strategies were nominated more often than active self-management strategies. Conventional medical strategies were the most common strategies utilised. Use of prescription or over-the-counter medication (most commonly paracetamol and ibuprofen), physiotherapy, and visiting a medical practitioner were the most commonly reported management techniques used. Cognitive strategies were not employed at all by this population and exercise as an active behavioural strategy was only used by a small percentage of female office workers with neck pain. It is apparent that most participants employed more than one strategy to manage their symptoms.

**Table 2:** Self-management strategies used by female office workers with neck pain (*n* = 51)

Strategy used	Individuals reporting % (n)
*Active Coping Strategies*	
*Behavioural:*	
Exercise	5.88 % (3)
Stretches	1.96 % (1)
Posture Modification	1.96 % (1)
*Cognitive:*	
Mental Distraction, relaxation	0 % (0)
*Passive Coping Strategies*	
*Behavioural:*	
Massage	11.76 % (6)
Aromatherapy	1.96 % (1)
*Conventional Medical:*	
Medication	82.35 % (42)
Physiotherapy	64.71 % (33)
General Practitioner	54.90 % (28)
Chiropractor	19.61 % (10)
Medical Specialist	13.73 % (7)
Other health care practitioner	7.84 % (4)
Acupuncture	3.92 % (2)

Table 3 shows the categories and combination of self-management strategies used by female office workers with neck pain, the most common being the use of conventional medical management. Only five female office workers indicated not using anything to manage their pain and six used a combination of strategies.

**Table 3:** Combinations of self-management strategies used by female office workers to manage their neck pain (*n* = 51)

Management combinations	Percent	(n)
Conventional medical only	70.59 %	(36)
No management	9.80 %	(5)
Passive behavioural & conventional medical	7.84 %	(4)
Active behavioural only	5.88 %	(3)
Passive behavioural only	3.92 %	(2)
Active behavioural, passive behavioural & conventional medical	1.96 %	(1)
Active behavioural & passive behavioural	0.00 %	(0)
Active behavioural & conventional medical	0.00 %	(0)
Cognitive only	0.00 %	(0)

## Discussion

This study identified that the severity of neck pain in female office workers is mild but that it has negative impact on their work and leisure time activity. Approximately half of the sample reported that their participation in usual activities of daily living was reduced due to their neck pain. The number of workers compensations claims submitted was low with most participants using passive coping strategies to manage their pain and remain at work. The significant impact of neck pain on function has been highlighted by other researchers [4, 28, 29].

Nearly half of the participants nominated computer work or the ergonomic environment at work as the source of their neck pain. While there is ample evidence linking musculoskeletal symptoms of the neck with computer use or features of the ergonomic workstation [6, 30–32], causation cannot be inferred in this cross-sectional study. Prospective studies and systematic reviews offer mixed evidence for the relationship between the ergonomic environment and incident neck pain. Côté et al. [33] found that poor computer workstation design and work posture were two of several factors associated with the development of an episode of neck pain. In contrast, another systematic review found strong evidence that high keyboard usage time and poor perception of computer placement have no predictive value for the onset of neck pain [34].

It is interesting to note that 22.4 % of the participants identified that their work activity had decreased. This is similar to two previous studies which also identified self-perceived reductions in productivity at work due to neck problems of 26 % in the Netherlands [35] and 13 % in Sweden [36]. Our study is the first in Australia to suggest the possibility of reduced productivity due to neck problems in office workers. This is an important finding as it suggests that “presenteeism” may be a concern in the workplace. Presenteeism has been defined as the decrease in performance associated with a worker remaining at work whilst impaired by a health condition [37]. Although challenging to measure, it has been estimated that presenteeism can result in up to four times greater loss in productivity than absenteeism [37, 38]. Thus, the cost of presenteeism for the workplace may be greater than direct health care costs [39, 40]. Our study suggests that this may be occurring in office workers with neck pain and requires further investigation.

The sub-sample of office workers used a range of self-management strategies, mostly passive in nature. This is consistent with findings in other neck pain related studies [41]. Passive coping strategies were reported more often than active strategies in our sub-sample of female office workers and usually consisted of over the counter or prescription medications, physiotherapy and/or consultation with a general practitioner.

It is surprising that exercise was reported by so few participants as a strategy to manage neck pain. Two systematic reviews have found strong evidence to support the positive effect of muscle strengthening and endurance exercises for controlling neck pain in office workers [42, 43]. When combined with manual therapy, these interventions, which consist of both passive and active strategies, produce greater improvements in pain, function, quality of life and patient satisfaction compared to manipulation or mobilisation alone for chronic neck pain [44]. Health practitioners are encouraged to recommend such interventions for office workers with neck pain.

There is evidence that the use of active self-management strategies can substantially reduce the likelihood of developing disabling pain [27, 45, 46]. Conversely, the use of passive coping strategies has been associated with an increased risk of developing disabling trouble or pain related disability [35, 45, 47]. Carroll et al. [41] identified that high use of passive coping strategies to manage neck or lower back pain can lead to the inability to work or carry out usual activities of daily living. This is reflected in our study, where passive coping strategies were mainly used, and could explain why neck pain had a significant impact on leisure activities, activities of daily living and work activity. Hence, it would be useful for clinicians to include education on active self-management strategies for neck pain. Recently, active self-management strategies have been shown to be more effective than passive physiotherapy techniques by increasing self-efficacy [46].

In our sample, only a small proportion submitted a workers’ compensation claim for their neck pain supporting our primary hypothesis. There are several reasons for the lack of claims submitted for neck problems in the working population. Firstly, neck pain is common in the general population [48] thus it would be difficult to establish the relative contribution of work. Secondly, the neck pain may be effectively managed with conservative treatments and of insufficient severity and duration to warrant the trouble of submitting a claim.

### Study limitations

The conclusions drawn from this study need to be considered in light of several limitations. The findings of this study cannot be generalised beyond female office workers. This sample may not be a true representation of office workers as many potential participants may have already left the workplace or sought alternate employment due to neck pain. As only 29 % of the study sample agreed to attend for further assessment of their neck pain, it is possible that selection bias contributed to the interview findings of the 51 office workers with only those with significant pain attending. Thus the results of this research cannot be generalized to all female office workers with neck pain. However, the level of neck pain and disability of this subsample did not differ from the sample population suggesting their responses were representative of the larger sample of office workers. While a strength of this study was the use of validated tools to measure health outcomes, they were based on self-reports rather than objective measures. This has been linked with both over and under estimation of prevalence of health outcomes [49, 50]. It is possible that measurement error was introduced due to common method bias or information bias (e.g. item characteristics or context), however the magnitude is thought to be small [51]. Attempts were made to limit method bias. For example, during the interview, the researcher used open-ended questions to determine participant’s perception of the source of their neck pain or the strategies used to manage it. Self-report measures of work absence and workers’ compensation data were not validated with employee records due to lack of access to these databases. However, the use of questionnaires to establish rates of sickness absences is considered a valuable source of information correlating relatively well with company records [52]. Despite the limitations identified, the findings of this research provide insight into the self-reported impact on neck pain in a working population of female office workers.

## Conclusion

This study has important implications for the workplace and health professionals. Although the severity of neck pain in this sample of female office workers is low, the impact on work is of concern. This study suggests the level of presenteeism in the female office workers may be significant, with many self-managing by reducing work and/or leisure activity and utilizing passive coping strategies. Future research should investigate the benefits of active coping strategies for office workers with neck problems to the individual and employer. Physiotherapists are well placed to assist office workers self-manage their symptoms to ensure they remain healthy at work.

## Abbreviations

NDI, neck disability index; NMQ, nordic musculoskeletal questionnaire
